# Indium–Tin–Oxide Nanostructures for Plasmon-Enhanced Infrared Spectroscopy: A Numerical Study

**DOI:** 10.3390/mi10040241

**Published:** 2019-04-11

**Authors:** Zhangbo Li, Zhiliang Zhang, Kai Chen

**Affiliations:** Institute of Photonics Technology, Jinan University, Guangzhou 511443, China; zhangboli_jnu@163.com (Z.L.); zhi_liang_zhang@163.com (Z.Z.)

**Keywords:** indium–tin oxide (ITO), plasmonics, nanoantenna, infrared spectroscopy

## Abstract

Plasmonic nanoantennas can significantly enhance the light–matter interactions at the nanoscale, and as a result have been used in a variety of applications such as sensing molecular vibrations in the infrared range. Indium–tin–oxide (ITO) shows metallic behavior in the infrared range, and can be used for alternative plasmonic materials. In this work, we numerically studied the optical properties of hexagonal ITO nanodisk and nanohole arrays in the mid-infrared. Field enhancement up to 10 times is observed in the simulated ITO nanostructures. Furthermore, we demonstrated the sensing of the surface phonon polariton from a 2-nm thick SiO_2_ layer under the ITO disk arrays. Such periodic arrays can be readily fabricated by colloidal lithography and dry etching techniques; thus, the results shown here can help design efficient ITO nanostructures for plasmonic infrared applications.

## 1. Introduction

Infrared spectroscopy is a traditional characterization technique for the accurate detection of chemical and biological species thanks to the characteristic absorption bands of the consisting chemical bonds, which form the “fingerprints” of the molecules. However, it is facing increasing difficulty when the amount of the analytes becomes less and less down to the level of monolayers or a few molecules because of the extremely small absorption cross-sections of the molecules. Surface-enhanced infrared absorption (SEIRA) spectroscopy is an enabling technique that can dramatically increase the absorption cross-sections of the molecules with significantly enhanced near-field intensities [1,2,3,4,5,6,7]. In general, the SEIRA substrates employ rough surfaces, metallic nanoparticles, or lithographically-defined nanostructures. Due to the lighting-rod effect and the excitation of surface plasmons, metallic nanostructures, which are usually made from Au or Ag, afford enormous near-field enhancement around the nanostructure surfaces [8,9,10]. The plasmon resonances of the nanoparticles can be readily tuned by controlling the shape, material, and environment of the nanoparticles [11,12,13]. In particular, periodic nanoparticle arrays can be achieved by e-beam lithography (EBL) or focused-ion beam (FIB), providing better control over the spectral positions of the plasmon resonances that are preferred to overlap with the molecular vibrations for enhanced sensitivity [14,15]. Besides nanoparticle-based SEIRA, there is another class of SEIRA that employs tip-based nano-IR systems and has achieved tremendous progress recently [16,17,18]. Such systems take advantage of the sharp-tip-induced large field enhancement to achieve ultrasensitive molecular detection with both high spectral and spatial resolution enabling promising applications for IR spectroscopy [19,20]. 

Traditionally, the noble metal Au is widely used as the plasmonic material due to its chemical stability, biocompatibility, and the ease of surface functionalization, which is particularly advantageous for biosensing applications. However, Au suffers from high intrinsic loss and an increasing price, which hampers its practical applications. Therefore, alternative plasmonic materials have been pursued by researchers [21,22,23], and light metal aluminum (Al) has been revisited as an alternative plasmonic material in the visible as well as in the infrared range [24,25]. The 2 to 3-nm thick natural oxide layer (Al_2_O_3_) can not only protect the underneath Al metal from oxidation, but also provide a means for stable surface functionalization [26,27,28,29]. Heavily doped oxide materials such as transparent conducting oxides (TCO) offer another option for low-loss, complementary metal-oxide-semiconductor (CMOS)-compatible plasmonic materials in the infrared range [30,31,32,33,34,35], among which indium–tin–oxide (ITO) is widely studied. ITO nanostructures have been demonstrated for refractive sensing [30] and SEIRA [32,36,37,38]. These ITO nanostructures are usually fabricated by top–down lithography or complex growth mechanisms; hence, more cost-effective and facile nanofabrication techniques are preferred. Colloidal lithography that employs monolayers of polymer spheres as deposition or etching masks provides an efficient method to fabricate various nanoparticle arrays [27,39,40]. In this work, we numerically studied the optical properties of ITO nanodisks and nanoholes that can be fabricated by colloidal lithography. Furthermore, we studied the coupling between the plasmon modes of the ITO nanoparticles and the surface phonon polaritons in a thin SiO_2_ layer. The results shown here can serve as a guideline for future experimental studies that use ITO for infrared plasmonic sensing applications.

## 2. Methods

**Figure 1:**
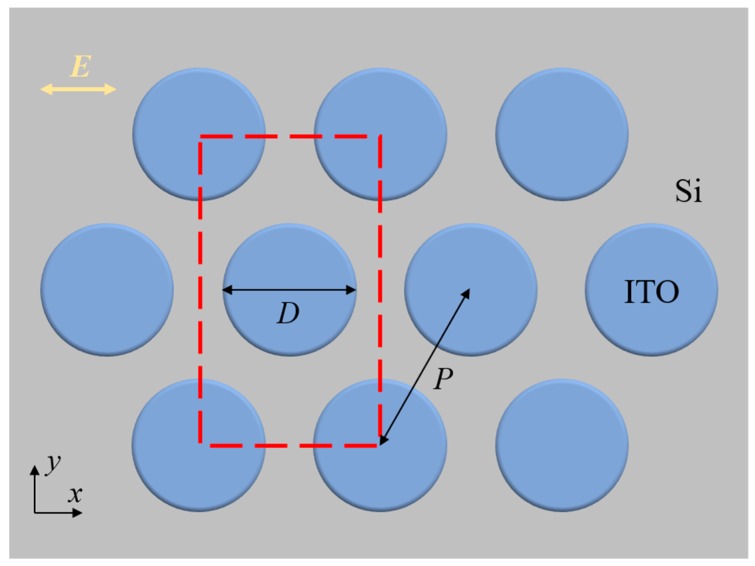
Illustration of the indium–tin–oxide (ITO) nanodisk array and the numerical simulation region. The ITO nanodisks with diameter D are arranged in a hexagonal array on a Si substrate. The red dashed line indicates the unit cell in the simulation, and the incident light is polarized in the *x* direction.

Figure 1 shows the geometrical configuration of a representative sample of the studied ITO nanodisk array. The ITO nanodisks sit on top of a Si substrate and form a hexagonal array. Such periodic structures can be fabricated using colloidal lithography and dry etching techniques at a large scale, where the polystyrene spheres act as etching masks for the underneath ITO layers. The diameter of the disks, D, can be precisely controlled by the etching time, providing a facile method of fabricating uniform large-area disk arrays. The rectangle defined by the red dashed line represents the unit cell in the finite time domain difference (FDTD) numerical simulations. Periodic boundary conditions are applied in the *x* and *y* directions, while a perfectly matched layer (PML) boundary condition is used in the *z* direction. A 2-nm fine mesh is applied in the regions with ITO microdisks. The incident light is polarized in the *x* direction and propagates in the *z* direction. The reflected light is collected to characterize the optical properties of the ITO disk array. The dielectric constants of Si are obtained from the handbook by Palik [41]. The dielectric constants of ITO are obtained from the Ref. 42 [42].

## 3. Results and Discussions

**Figure 2:**
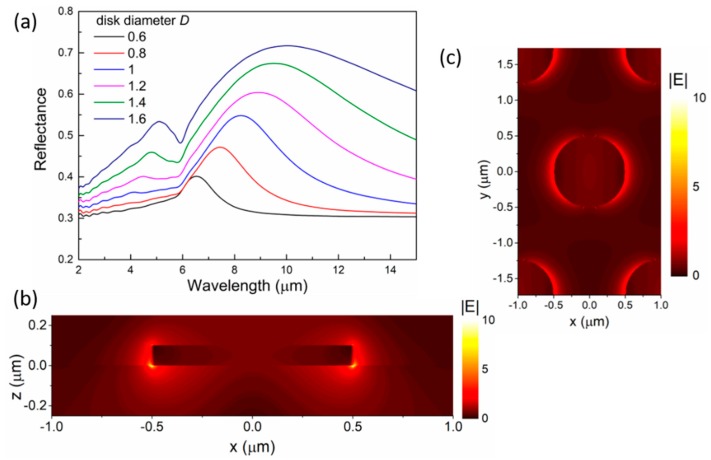
Optical properties of an ITO disk array with P = 2 μm. (**a**) The reflectance spectra of the ITO disks with different diameters. (**b**) The electric near-field profile inside the xz cross-section at 8.24 μm for the disk with D = 1 μm. The enhanced field is concentrated near the disk edges and close to the Si substrate. (**c**) The electric near-field profile of the disk array as in (**b**) at 8.24 μm. The magnitude of the electric field is plotted in panels (**b**) and (**c**).

For disk arrays fabricated by colloidal lithography, the periodicity P of the arrays is determined by the diameter of the original polystyrene spheres used as etching masks. Thus, in our simulations, we choose the value of P according to some commercially available microspheres, namely P = 2 μm, 3 μm, and 4.4 μm, and the thickness of the disks is fixed at 100 nm. Figure 2a shows the effect of the disk diameter on the reflectance spectra of the samples. As the most popular TCO material, ITO displays metallic behavior in the infrared wavelength range. The real part (ε_1_) of the dielectric constants of the ITO (obtained from Ref. 42) crosses zero at 1.6 μm from the positive to the negative region. The reflectance spectra of the disk arrays show similar trends to those of metallic disk arrays. As the disk diameter D increases, the resonance peak shifts to the longer wavelength range, and the peak intensity increases at the same time. Figure 2b,c show the near-field distribution of the electric field at 8.24 μm for the disk array with D = 1 μm and P = 2 μm. Clearly, the near field shows enhancement around the disk edges at resonance wavelengths, indicating the dipolar character of the plasmon resonance. As shown in Figure 2b, the hot spots are located at the ITO/Si interface. 

**Figure 3:**
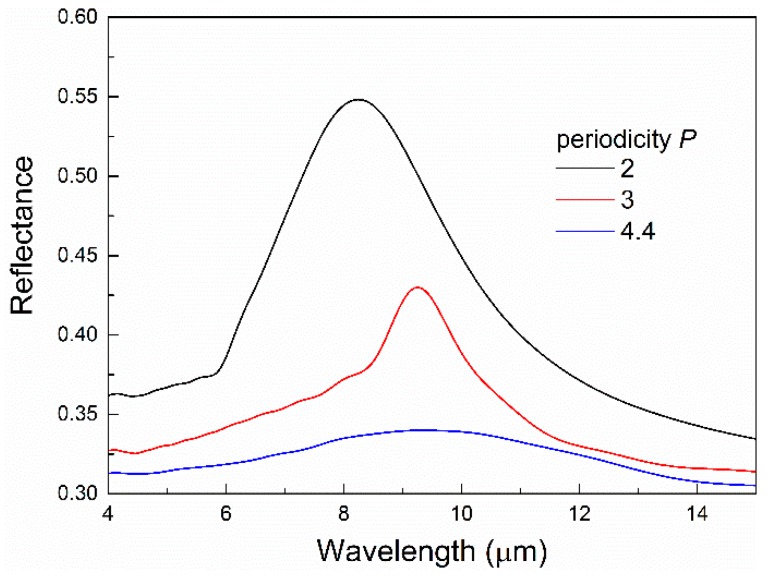
The effect of the periodicity P on the reflectance spectra of the disk arrays with D = 1 μm.

Figure 3 shows the effect of the periodicity of the array on the reflectance spectra. The diameter of the disks is fixed at 1 μm. With large periodicity, the filling factor of the disk arrays is low, and thus a broad and shallow peak is observed. As the periodicity becomes smaller, the disk density becomes larger, and more light is reflected and scattered. It is noted that lattice coupling exists inside a nanoparticle array, and the coupling strength depends on the array geometry, particularly the period. At a certain period, constructive interference occurs, leading to a much narrower resonance peak [2]. In our case, such constructive interference happens when the period is ~3 μm, and therefore, smaller or larger periods result in broader resonance peaks.

**Figure 4:**
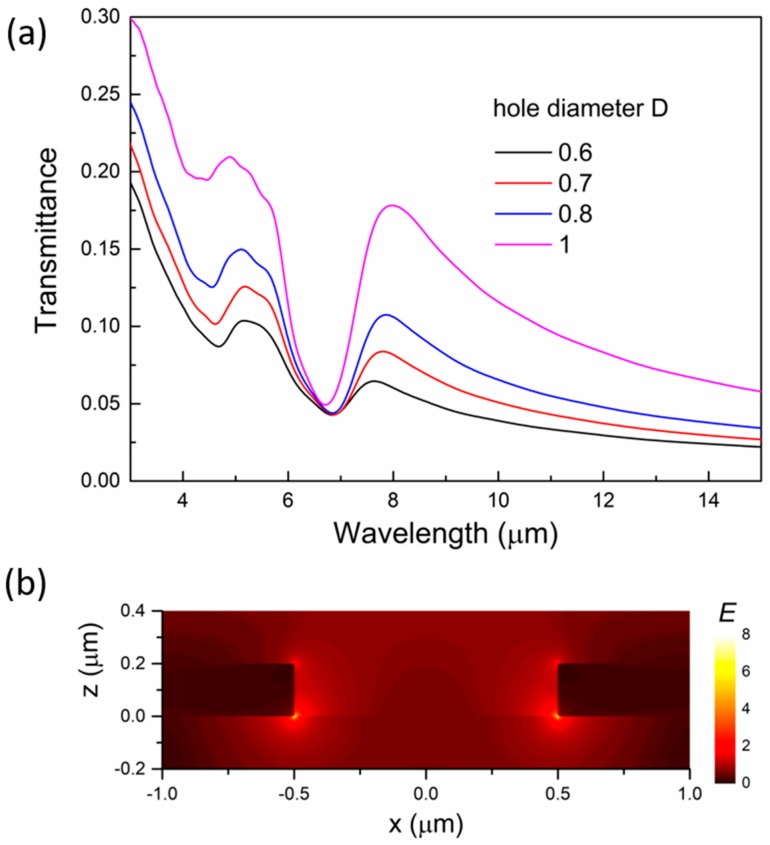
The optical properties of ITO nanohole arrays. (**a**) The reflectance spectra of the ITO nanohole arrays with different hole diameters. The periodicity of the arrays is 2 μm. (**b**) The electric near-field distribution of the nanohole at 8 μm. The diameter of the nanohole is 1 μm, and the color bar shows the magnitude of the electric field.

The complementary nanostructures to the disk arrays, i.e., the hexagonal nanohole arrays, can also be easily fabricated by colloidal lithography, where size-reduced spheres can be used as deposition masks for ITO. Therefore, we also numerically studied ITO nanohole arrays, and the results are shown in Figure 4. Similar to the disk arrays in Figure 2, the periodicity of the nanohole arrays is fixed at 2 μm, and the thickness is fixed at 200 nm. We only vary the diameters of the nanoholes. Nanohole arrays are known to support extraordinary optical transmission (EOT) due to the excitation of the localized surface plasmon modes of the nanoholes. A major EOT peak is observed in the transmittance spectra at ~8 μm. As the hole diameter increases, the peak intensity increases while the peak position stays in the vicinity of 8 μm in contrast to the large red-shift observed in the ITO disk arrays, as shown in Figure 2a. The different optical behavior can be attributed to the complex mechanism behind the EOT phenomenon where the transmission is mediated by the localized plasmon mode of individual nanoholes and the collective optical modes of the arrays [43,44]. Figure 4b displays the near-field distribution of the electric field at the resonance peak (8 μm).

It is noted that the free carrier density of ITO depends on the preparation techniques as well as post-deposition annealing [42,45]. Thus, the dielectric constant of ITO can be readily tuned via various parameters in deposition and annealing, providing an effective means of controlling the optical properties of ITO nanoantennas and making ITO an attractive material for infrared plasmonics [30]. The results presented here are based on the dielectric constants in earlier reports [42].

**Figure 5:**
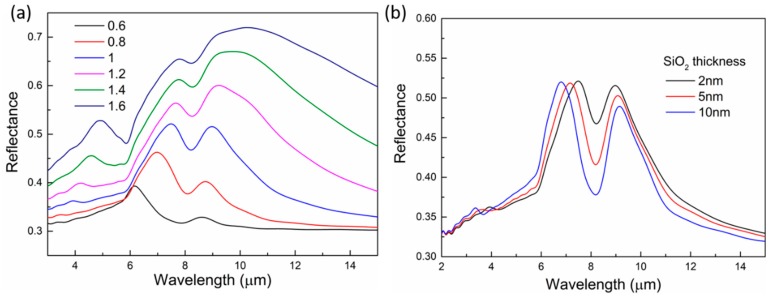
Sensing of the surface phonon polariton from SiO_2_ with ITO nanodisks. (**a**) Reflectance spectra of the ITO disk arrays with a 2-nm thick SiO_2_ layer underneath the arrays. The diameter of the disks is changed to tune the plasmon resonances and study the coupling between the plasmon mode and the SiO_2_ vibration. (**b**) Reflectance spectra of the disk array (P = 2 μm and D = 1 μm) with different SiO_2_ layer thickness.

The infrared nanoantennas can concentrate the electromagnetic field into subwavelength volume, leading to significantly enhanced near-field intensity, i.e., hot spots, which have been utilized in various SEIRA reports with different nanoantenna designs and materials, including TCO materials [46]. As we shown in Figure 2, such near-field enhancement occurs around the ITO disks. We further investigated their sensing capability by placing a 2-nm thick SiO_2_ layer under the ITO disks and detecting the surface phonon polariton (SPhP) signal from the SiO_2_. The collected reflectance spectra are shown in Figure 5a, where the simulated disk arrays have the same dimensions as those in Figure 2a. Compared with Figure 2a, distinct dips are observed on these spectra, and the dips appear around 8 μm, which is inside the range of the Fuchs–Kliewer surface phonon polariton of SiO_2_, and is consistent with previous reports [40]. It is noted that the presence of ITO microdisks facilitate the excitation of the SiO_2_ SPhP, as the required additional momentum could be provided by the scattered light from the microdisks [47]. The shape and intensity of the dip are apparently affected by the detuning between the vibrations and the plasmon resonances. When the plasmon peak shows a good overlap with the vibration, as for ITO disks with D = 1 μm, the intensity of the dip reaches the maximum. Thus, we chose this ITO disk array (P = 2 μm and D = 1 μm) to study the effect of the SiO_2_ layer thickness, as shown in Figure 5b. As the SiO_2_ layer thickness increases, the intensity of the dip increases, because more SiO_2_ is included inside the volume of the hot spots, resulting in a stronger coupling between the plasmon mode and the vibration. 

## 4. Conclusions

In summary, we have numerically studied the optical properties of hexagonal ITO nanodisk arrays and nanohole arrays. We specifically tune the dimensions of the nanostructures to shift their plasmon resonances in the infrared wavelength range, making them suitable for SEIRA sensing applications. Both the ITO disks and nanoholes show distinct plasmon resonances in the mid-infrared range, and field enhancement up to 10 times is observed in the ITO nanostructures. The resonances of the ITO disk arrays can be readily tuned in a broad wavelength range. We also demonstrate the excitation and sensing of the surface phonon polariton signal from a 2-nm thick SiO_2_ layer under the disk arrays. Both the ITO nanodisk and nanohole arrays can be fabricated on a large scale using colloidal lithography and dry etching methods. Thus, the results shown here can be useful for the design and application of ITO nanostructures operating in the mid-infrared wavelength range.

## References

[1] Neubrech F., Pucci A., Cornelius T.W., Karim S., Garcia-Etxarri A., Aizpurua J. (2008). Resonant Plasmonic and Vibrational Coupling in a Tailored Nanoantenna for Infrared Detection. Phys. Rev. Lett..

[2] Adato R., Yanik A.A., Amsden J.J., Kaplan D.L., Omenetto F.G., Hong M.K., Erramilli S., Altug H. (2009). Ultra-sensitive vibrational spectroscopy of protein monolayers with plasmonic nanoantenna arrays. Proc. Natl. Acad. Sci. USA.

[3] Wu C., Khanikaev A.B., Adato R., Arju N., Yanik A.A., Altug H., Shvets G. (2012). Fano-resonant asymmetric metamaterials for ultrasensitive spectroscopy and identification of molecular monolayers. Nat. Mater..

[4] Chen K., Adato R., Altug H. (2012). Dual-Band Perfect Absorber for Multispectral Plasmon-Enhanced Infrared Spectroscopy. ACS Nano.

[5] Hoang C.V., Oyama M., Saito O., Aono M., Nagao T. (2013). Monitoring the Presence of Ionic Mercury in Environmental Water by Plasmon-Enhanced Infrared Spectroscopy. Sci. Rep..

[6] Cetin A.E., Etezadi D., Altug H. (2014). Accessible Nearfields by Nanoantennas on Nanopedestals for Ultrasensitive Vibrational Spectroscopy. Adv. Opt. Mater..

[7] Bagheri S., Weber K., Gissibl T., Weiss T., Neubrech F., Giessen H. (2015). Fabrication of Square-Centimeter Plasmonic Nanoantenna Arrays by Femtosecond Direct Laser Writing Lithography: Effects of Collective Excitations on SEIRA Enhancement. ACS Photonics.

[8] Neubrech F., Weber D., Katzmann J., Huck C., Toma A., Di Fabrizio E., Pucci A., Härtling T. (2012). Infrared Optical Properties of Nanoantenna Dimers with Photochemically Narrowed Gaps in the 5 nm Regime. ACS Nano.

[9] Hoffmann J.M., Janssen H., Chigrin D.N., Taubner T. (2014). Enhanced infrared spectroscopy using small-gap antennas prepared with two-step evaporation nanosphere lithography. Opt. Express.

[10] Enders D., Nagao T., Pucci A., Nakayama T., Aono M. (2011). Surface-enhanced ATR-IR spectroscopy with interface-grown plasmonic gold-island films near the percolation threshold. Phys. Chem. Chem. Phys..

[11] Gaspar D., Pimentel A.C., Mateus T., Leitão J.P., Soares J., Falcão B.P., Araújo A., Vicente A., Filonovich S.A., Águas H. (2013). Influence of the layer thickness in plasmonic gold nanoparticles produced by thermal evaporation. Sci. Rep..

[12] Chen K., Leong Eunice Sok P., Rukavina M., Nagao T., Liu Yan J., Zheng Y. (2015). Active molecular plasmonics: Tuning surface plasmon resonances by exploiting molecular dimensions. Nanophotonics.

[13] Araújo A., Mendes M.J., Mateus T., Vicente A., Nunes D., Calmeiro T., Fortunato E., Águas H., Martins R. (2016). Influence of the Substrate on the Morphology of Self-Assembled Silver Nanoparticles by Rapid Thermal Annealing. J. Phys. Chem. C.

[14] Maß T.W.W., Taubner T. (2015). Incident Angle-Tuning of Infrared Antenna Array Resonances for Molecular Sensing. ACS Photonics.

[15] Weber D., Albella P., Alonso-González P., Neubrech F., Gui H., Nagao T., Hillenbrand R., Aizpurua J., Pucci A. (2011). Longitudinal and transverse coupling in infrared gold nanoantenna arrays: Long range versus short range interaction regimes. Opt. Express.

[16] Chen J., Badioli M., Alonso-Gonzalez P., Thongrattanasiri S., Huth F., Osmond J., Spasenovic M., Centeno A., Pesquera A., Godignon P. (2012). Optical nano-imaging of gate-tunable graphene plasmons. Nature.

[17] Alonso-González P., Albella P., Schnell M., Chen J., Huth F., García-Etxarri A., Casanova F., Golmar F., Arzubiaga L., Hueso L.E. (2012). Resolving the electromagnetic mechanism of surface-enhanced light scattering at single hot spots. Nat. Commun..

[18] Taubner T., Eilmann F., Hillenbrand R. (2005). Nanoscale-resolved subsurface imaging by scattering-type near-field optical microscopy. Opt. Express.

[19] Lewin M., Baeumer C., Gunkel F., Schwedt A., Gaussmann F., Wueppen J., Meuffels P., Jungbluth B., Mayer J., Dittmann R. (2018). Nanospectroscopy of Infrared Phonon Resonance Enables Local Quantification of Electronic Properties in Doped SrTiO_3_ Ceramics. Adv. Funct. Mater..

[20] Xu X.G., Rang M., Craig I.M., Raschke M.B. (2012). Pushing the Sample-Size Limit of Infrared Vibrational Nanospectroscopy: From Monolayer toward Single Molecule Sensitivity. J. Phys. Chem. Lett..

[21] West P.R., Ishii S., Naik G.V., Emani N.K., Shalaev V.M., Boltasseva A. (2010). Searching for better plasmonic materials. Laser Photonics Rev..

[22] Kumar M., Umezawa N., Ishii S., Nagao T. (2016). Examining the Performance of Refractory Conductive Ceramics as Plasmonic Materials: A Theoretical Approach. ACS Photonics.

[23] Yang X., Sun Z., Low T., Hu H., Guo X., García de Abajo F.J., Avouris P., Dai Q. (2018). Nanomaterial-Based Plasmon-Enhanced Infrared Spectroscopy. Adv. Mater..

[24] Knight M.W., King N.S., Liu L., Everitt H.O., Nordlander P., Halas N.J. (2013). Aluminum for Plasmonics. ACS Nano.

[25] Lecarme O., Sun Q., Ueno K., Misawa H. (2014). Robust and Versatile Light Absorption at Near-Infrared Wavelengths by Plasmonic Aluminum Nanorods. ACS Photonics.

[26] Cerjan B., Yang X., Nordlander P., Halas N.J. (2016). Asymmetric Aluminum Antennas for Self-Calibrating Surface-Enhanced Infrared Absorption Spectroscopy. ACS Photonics.

[27] Chen K., Dao T.D., Ishii S., Aono M., Nagao T. (2015). Infrared Aluminum Metamaterial Perfect Absorbers for Plasmon-Enhanced Infrared Spectroscopy. Adv. Funct. Mater..

[28] Ayas S., Topal A.E., Cupallari A., Güner H., Bakan G., Dana A. (2014). Exploiting Native Al_2_O_3_ for Multispectral Aluminum Plasmonics. ACS Photonics.

[29] Canalejas-Tejero V., Herranz S., Bellingham A., Moreno-Bondi M.C., Barrios C.A. (2014). Passivated aluminum nanohole arrays for label-free biosensing applications. ACS Appl. Mater. Interfaces.

[30] Li S.Q., Guo P., Zhang L., Zhou W., Odom T.W., Seideman T., Ketterson J.B., Chang R.P.H. (2011). Infrared Plasmonics with Indium–Tin-Oxide Nanorod Arrays. ACS Nano.

[31] Babicheva V.E., Kinsey N., Naik G.V., Ferrera M., Lavrinenko A.V., Shalaev V.M., Boltasseva A. (2013). Towards CMOS-compatible nanophotonics: Ultra-compact modulators using alternative plasmonic materials. Opt. Express.

[32] Abb M., Wang Y., Papasimakis N., de Groot C.H., Muskens O.L. (2014). Surface-Enhanced Infrared Spectroscopy Using Metal Oxide Plasmonic Antenna Arrays. Nano Lett..

[33] Kim J., Dutta A., Memarzadeh B., Kildishev A.V., Mosallaei H., Boltasseva A. (2015). Zinc Oxide Based Plasmonic Multilayer Resonator: Localized and Gap Surface Plasmon in the Infrared. ACS Photonics.

[34] Guo P., Schaller R.D., Ketterson J.B., Chang R.P.H. (2016). Ultrafast switching of tunable infrared plasmons in indium tin oxide nanorod arrays with large absolute amplitude. Nat. Photonics.

[35] Wang Y., Overvig A.C., Shrestha S., Zhang R., Wang R., Yu N., Dal Negro L. (2017). Tunability of indium tin oxide materials for mid-infrared plasmonics applications. Opt. Mater. Express.

[36] Chen K., Guo P., Dao T.D., Li S.-Q., Ishiii S., Nagao T., Chang R.P.H. (2017). Protein-Functionalized Indium-Tin Oxide Nanoantenna Arrays for Selective Infrared Biosensing. Adv. Opt. Mater..

[37] Kamakura R., Takeishi T., Murai S., Fujita K., Tanaka K. (2018). Surface-Enhanced Infrared Absorption for the Periodic Array of Indium Tin Oxide and Gold Microdiscs: Effect of in-Plane Light Diffraction. ACS Photonics.

[38] D’apuzzo F., Esposito M., Cuscunà M., Cannavale A., Gambino S., Lio G.E., De Luca A., Gigli G., Lupi S. (2018). Mid-Infrared Plasmonic Excitation in Indium Tin Oxide Microhole Arrays. ACS Photonics.

[39] Dao T.D., Chen K., Ishii S., Ohi A., Nabatame T., Kitajima M., Nagao T. (2015). Infrared Perfect Absorbers Fabricated by Colloidal Mask Etching of Al–Al_2_O_3_–Al Trilayers. ACS Photonics.

[40] Chen K., Duy Dao T., Nagao T. (2017). Tunable Nanoantennas for Surface Enhanced Infrared Absorption Spectroscopy by Colloidal Lithography and Post-Fabrication Etching. Sci. Rep..

[41] Palik E.D. (1998). Handbook of Optical Constants of Solids.

[42] Tamanai A., Dao T.D., Sendner M., Nagao T., Pucci A. (2017). Mid-infrared optical and electrical properties of indium tin oxide films. Phys. Status Solidi A.

[43] Garcia-Vidal F.J., Martin-Moreno L., Ebbesen T.W., Kuipers L. (2010). Light passing through subwavelength apertures. Rev. Mod. Phys..

[44] Laux E., Genet C., Ebbesen T.W. (2009). Enhanced optical transmission at the cutoff transition. Opt. Express.

[45] Baía I., Quintela M., Mendes L., Nunes P., Martins R. (1999). Performances exhibited by large area ITO layers produced by r.f. magnetron sputtering. Thin Solid Films.

[46] Neubrech F., Huck C., Weber K., Pucci A., Giessen H. (2017). Surface-Enhanced Infrared Spectroscopy Using Resonant Nanoantennas. Chem. Rev..

[47] Neubrech F., Weber D., Enders D., Nagao T., Pucci A. (2010). Antenna Sensing of Surface Phonon Polaritons. J. Phys. Chem. C.

